# Prevalence of Transient Osteoporosis of the Hip Among Patients Presenting With Hip Pain in a Major Tertiary Hospital in Saudi Arabia

**DOI:** 10.7759/cureus.30875

**Published:** 2022-10-30

**Authors:** Asim S Aldhilan, Salma O Al-Amoudi, Sarah S Baabbad, Hamad M Al Jubair, Abdulmalik B Albaker, Yassir Edrees Almalki, Ali A Alamer, Sharifa Alduraibi, Ziyad A Almushayti, Suhayb Aldhilan

**Affiliations:** 1 Department of Radiology, College of Medicine, Qassim University, Buraydah, SAU; 2 Department of Musculoskeletal Imaging and Diagnostic Radiology, King Faisal Specialist Hospital and Research Center, Riyadh, SAU; 3 Department of Musculoskeletal Imaging, Radio Diagnostics and Medical Imaging, Prince Sultan Military Medical City, Riyadh, SAU; 4 Department of Musculoskeletal Imaging, Radiodiagnostics and Medical Imaging, Prince Sultan Military Medical City, Riyadh, SAU; 5 Department of Orthopedics, College of Medicine, Majmaah University, Majmaah, SAU; 6 Division of Radiology, Department of Internal Medicine, Medical College, Najran University, Najran, SAU; 7 Department of Radiology, Qassim University, Buraydah, SAU; 8 Department of Radio Diagnostics and Medical Imaging, Prince Sultan Military Medical City, Riyadh, SAU

**Keywords:** saudi arabia, prevalence, mri, risk factors, transient osteoporosis of the hip

## Abstract

Background: Transient osteoporosis of the hip, or acute bone marrow edema syndrome, is a rare condition characterized by a decrease in bone mineral density of the proximal femur, which resolves with conservative management over 6-24 months. At presentation, the patient complains of sudden onset of localized pain in the hip, which is aggravated by weight-bearing. However, the prevalence and risk factors for this condition are still unclear.

Objective*:* This study aims to identify the prevalence of transient osteoporosis of the hip among patients who present with hip pain and underwent magnetic resonance imaging of the hip.

Method: This is a retrospective investigation that involved collecting data from patients' records in a tertiary hospital in Saudi Arabia. Included candidates were patients who presented with hip pain, had an MRI done between 2016 and 2019 inclusive, and were older than 14 years. The collected data involved the age and gender of patients, the hip's affected side, and the diagnosis. Data analysis was executed through SPSS version 26 (IBM Corp., Armonk, NY).

Results: Three hundred and fourteen patients matched our inclusion criteria. The prevalence of transient osteoporosis of the hip was 2.5%. All of them were males and half were above 40 years, 50% had pain in the left side, and 75% had a small joint effusion. The femoral head was the most affected part of the joint in patients with transient osteoporosis of the hip. Among our patients, the most common cause of hip pain was gluteus medius tendonitis (12.9%), where 33.1% of patients with hip pain had normal examination and investigations, and 15.2% had more than one condition. Risk factors for transient osteoporosis of the hip are pain in the left hip joint (p-value=0.023) and an age between 41 and 50 years (p-value=0.012).

Conclusion: The prevalence of transient osteoporosis of the hip is low, yet it requires confirmation by studies with a more robust design. Males older than 40 years and left-side hip pain are at higher risk.

## Introduction

Transient osteoporosis of the hip (TOH) is a clinical disease that affects the hip joint and is accompanied by significant pain and a reduction in patients’ quality of life [1]. The disease is benign and is also known as primary bone marrow emphysema syndrome [2]. The etiology of TOH is still unknown [3], though it can lead to significant complications, including avascular necrosis and hip fractures. The management of TOH is usually conservative, using medications [4]. However, several theories are put forth, which include microvascular injury, nontraumatic reflex sympathetic dystrophy, metabolic, viral infection, neurological, and endocrine factors [5].

Non-traumatic, sudden, or gradual onset pain in the groin, anterior thigh, or buttocks is the most common complaint. The pain is exacerbated by weight-bearing and worsens at night. TOH is known to be present in three phases. The first phase is characterized by a sudden onset of pain along with physical limitation. This phase lasts for one month. The second phase is a plateau phase; the signs and symptoms remain consistent over a period of one to two months with no worsening of symptoms. However, on radiographic examination, osteopenia is seen. The final phase is defined by the spontaneous regression of clinical features over a period of 4 months, and the bone density also returns to normalcy during this phase [6]. The prevalence of transient hip osteoporosis is thought to be higher in men, particularly those in their 40s [7]. It is thought to be significantly linked to pregnancy in women, especially during the third trimester. However, the incidence of fracture complications is higher in females, particularly during pregnancy [8]. A neurogenic hypothesis proposes that the compression of the mother’s obturator nerve by the head of the fetus causes TOH in such conditions [3].

A radiographic examination lags behind the clinical presentation, while a bone scan, although sensitive, is non-specific, thus it cannot be relied upon for a definitive diagnosis of TOH [3]. The gold standard for diagnosing TOH is magnetic resonance imaging (MRI) [9]. The diagnosis of TOH is made by low signal intensity for T1-weighted images and high signal intensity for T2-weighted images, in addition to a homogenous edematous pattern (the femoral head and neck) with a normal subchondral region [10]. It is also useful in differentiating TOH from avascular necrosis, insufficiency fractures, infections, and neoplasms. Based on the MRI findings, patients are started on medical treatment, including bisphosphonates, calcitonin, or teriparatide [11]. In the acute phase of the disease, traction has been found to be beneficial in both preventing and correcting the deformity due to joint effusion. Additionally, the range of hip movements could be retained with a physiotherapy regime with special emphasis on abductor muscle strengthening. Besides these therapies, surgical management in the form of core decompression has also been advocated. Additionally, a growing body of evidence suggests that hyperbaric oxygen therapy can also be considered an alternative therapy for pain management [3].

Despite the availability of TOH medications, some patients may still suffer from TOH complications [12]. These complications are commonly induced by a secondary factor such as ischemia, injury, neoplasm, medications, or surgery [13]. These factors can result in increased bone turnover or microfracture, which leads to edema. Accordingly, TOH can be primary or secondary, though this classification should consider a thorough assessment of TOH's risk factors [14]. Despite the abundance of information on the diagnosis and management of TOH in medical literature, data are scarce on TOH's prevalence and its risk factors, which deserve further exploration. Consequently, the present study evaluated TOH's prevalence and risk factors in a tertiary hospital in Saudi Arabia.

## Materials and methods

This retrospective study was carried out in a tertiary hospital in Saudi Arabia and included patients who presented with hip pain and had an MRI between 2016 and 2019. Inclusion criteria were patients older than 14 years without a medical illness that would explain the hip symptoms. Patients with a known diagnosis such as sickle cell disease, rheumatoid arthritis, or malignancy related to the hip area, and patients under 14 years were excluded.

**Figure 1:**
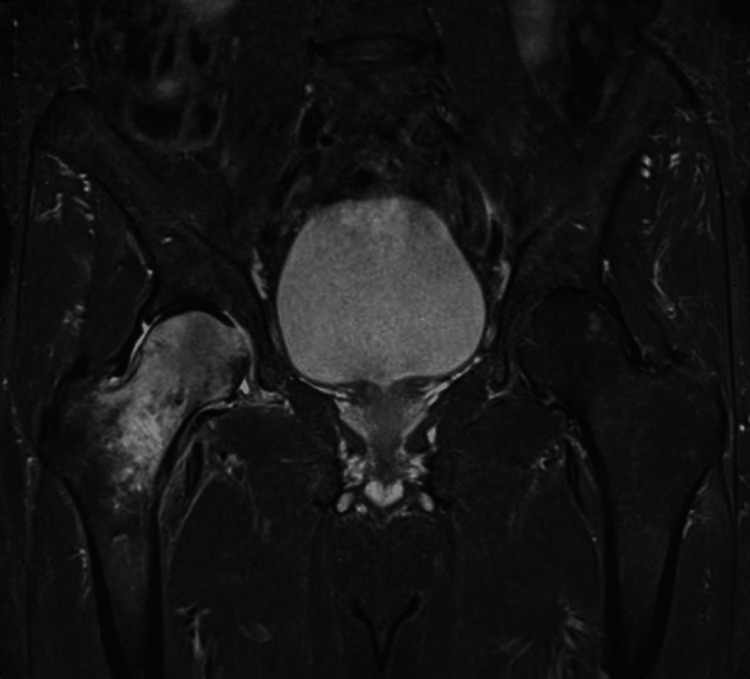
Hip osteoporosis image by magnetic resonance imaging

A pre-designed excel sheet was used for the data collection. Patients’ data were collected from patient records between 2016 and 2019, inclusive. The collected data involved patients’ gender and age, diagnosis, and the hip's affected side. Images were reviewed by two attendees who specialized in musculoskeletal imaging and were blinded to the final reports and had 100% agreeability in the positive cases for TOH (Figure 1).

Data were described in terms of frequencies and percentages for categorical variables, while means and standard deviations were used for numerical variables. Risk factors of TOH were compared using Chi-square testing at a level of significance p-value<0.05. The Statistical Package for the Social Sciences (SPSS) (version 26; IBM Corp., Armonk, NY) was used for data analysis. All the necessary ethical approvals were obtained from the ethical committee at Prince Sultan Military Medical City Scientific Research Centre with ref no. 1389, before the commencement of data collection from patients' records. The patient's identity was kept confidential throughout the study.

## Results

Three hundred and fourteen patients had hip pain over the study duration (between 2016 and 2019, inclusive). All descriptive and comparative analyses are shown below.

Demographics of patients

Among the 314 patients, only eight patients had TOH; all of them were males. The most prevalent age group among the whole cohort was between 41 and 50 years, with 27.7% in all patients, and 50% in patients with TOH, as shown in Table 1.

**Table 1:** Demographics of the included cohort n - number of patients

	All patients with hip pain (N=314)		Patients with TOH (N=8)	
	Count	Percent	Count	Percent
Gender				
Female	189	60.2	0	0
Male	125	39.8	8	100
Age				
15 to 20	9	2.9	0	0
21 to 30	36	11.5	1	12.5
31 to 40	74	23.6	1	12.5
41 to 50	87	27.7	4	50.0
51 to 60	60	19.1	1	12.5
More than 60	48	15.3	1	12.5

The affected side of the hip

Data about the affected side of the hip was collected. Of the whole cohort, 38.2% had pain in the right side of the hip, while for TOH patients, 50% had pain in the left side, as shown in Table 2.

**Table 2:** The affected side of the hip TOH - Transient osteoporosis of the hip, n - number of patients

	All patients with hip pain (N=314)		Patients with TOH (N=8)	
	Count	Percent	Count	Percent
Bilateral	54	17.2	0	0
Left	84	26.8	4	50.0
Right	120	38.2	3	37.5
Unspecified Hip pain	56	17.8	1	12.5

Other common causes of hip pain

Different diagnoses of patients were analyzed. TOH's prevalence was 2.5% among the whole cohort, while 33.1% of the patients who had hip pain had a normal MRI study. Around 15% of patients were diagnosed with more than one condition, while the most common single diagnosis was gluteus medius tendonitis, as shown in Table 3.

**Table 3:** Other common causes of hip pain

	Count	Percent
Normal	104	33.1
More than one condition	48	15.2
Gluteus Medius tendonitis	40	12.9
Osteoarthritis	13	4.1
Transient osteoporosis of the hip	8	2.5
Other Tendonitis	5	1.6
Avascular necrosis	5	1.6
Labral tear	4	1.3
Sacroiliitis	4	1.3
Trochanteric Bursitis	4	1.3
Gluteus Medius Tear	3	1.0
Enchondroma	2	0.6
Other	74	23.5

Post-hoc analysis for patients with TOH

Further analysis was carried out for patients with TOH. Dexa scan results were compared among the eight patients. Four patients did not have a DEXA scan, while the other four showed a Z score of the femur of -1.3, -1.5, -0.6, and -0.5, respectively, three in the left femur and one in the right.

Laboratory investigations for patients with TOH

Results of laboratory investigations for TOH patients were recorded. The average serum calcium level was 2.38±0.15, corrected calcium level 2.3±0.16, vitamin D 58.5±14.14, ESR 14.4±26.09, and average CRP level was 14.3±17.84, as shown in Table 4.

**Table 4:** Laboratory investigations for patients with TOH TOH - Transient osteoporosis of the hip, ESR - Erythrocyte sedimentation rate, CRP - C-reactive protein

	Count	Mean	Standard deviation	Minimum	Maximum	Reference values
Serum Calcium	7	2.38	0.15	2.23	2.64	2.25-2.62 mmol/L
Corrected Calcium	7	2.3	0.16	2.08	2.54	2.1 to 2.4 mmol/L
Vitamin D	7	58.5	14.14	43.2	79.7	25-80 ng/mL
ESR	5	14.4	26.09	1	61	< 22 mm/hr
CRP	4	14.3	17.84	5	41	<5 mg/L

Other characteristics of TOH patients

After following up on TOH patients, only one patient had a recurrence. None of the patients had previous episodes. As for the affected area of the joint, the femoral head-neck junction was the most commonly affected area seen in all of the eight patients with variations in extension as outlined in Table 5. Additionally, all of the patients had a small joint effusion.

**Table 5:** Other characteristics of TOH patients (N=8) N - number of patients, TOH - Transient osteoporosis of the hip

		Count	Percent
Recurrence		1	12.5
Previous episode		0	0
Affected part of the joint	Femur head and neck	4	50
	Femur head, neck, and greater trochanter	2	25
	Femur head, neck, and lesser troch	1	12.5
	Femur head, neck, intertrochanteric region	1	12.5
Joint effusion		8	100
Subchondral fracture		1	12.5

Risk factors of TOH

To identify TOH's risk factors, patients with and without TOH were compared over different categorical variables through Chi-square testing at a level of significance p-value<0.05. It has been demonstrated that pain in the left side of the hip (p-value=0.023), and aging between 41 and 50 years (p-value=0.012), represent the most significant risk factors for TOH.

## Discussion

TOH is characterized by severe pain in the hip area, usually confirmed by MRI [15]. Any inflammation or edema of the bones or joints can be diagnosed through MRI and then treated to reduce the pain [16]. Many risk factors have been identified for TOH in the medical literature. However, TOH's prevalence and risk factors in the Saudi population are yet to be explored [17].

The present investigation calculated TOH's prevalence and estimated its risk factor through a patient cohort with hip pain over four years in a tertiary hospital in Saudi Arabia. The study demonstrated that TOH's prevalence among Saudi adults is low and estimated to be 2.5%. The disease is more prevalent among males aged between 41 and 50 years with a left-side predominance. The majority of patients with a painful hip had a normal MRI study. Of those with abnormal MRI findings, the majority were found to have more than one underlying condition as the cause of their pain. On the other hand, the most prevalent single cause of hip pain was gluteus medius tendonitis (9.9%). Turning to TOH's risk factors, the left side of the hip (p-value=0.023) and aging between 41 and 50 years old (p-value=0.012) was the most significant risk factors for TOH in the Saudi population.

TOH has been examined in different contexts. Harsevoort et al. [18] examined the prevalence and risk factors of TOH in adult patients with congenital bone diseases, namely, osteogenesis imperfecta. Harsevoort et al. [18] included 314 patients retrospectively over 10 years and demonstrated a prevalence of TOH of 1.6%. However, Harsevoort et al. [18] could not identify any risk factors for TOH except the osteogenesis imperfecta. Balakrishnan A. et. al conducted a study at St. Michael's Hospital, University of Toronto. and they emphasized the fact that TOH is often misdiagnosed as avascular necrosis of the neck (AVN). They reviewed retrospectively 196 patients diagnosed with AVN between 1998 and 2001 and posted for surgery. Of these 196 patients, 10 patients with clinical features suggestive of TOH were identified. The diagnosis of TOH was confirmed once MRI was done and both the symptoms and MRI findings spontaneously resolved after 5.5 and 7.5 months, respectively. A greater level of awareness is required to avoid unnecessary surgical interventions [19]. The misdiagnosis is supported by the fact that there is a delay of six weeks to two months in diagnosing TOH patients [13,20].

Similarly, the prevalence of TOH in the present study was low (2.5%). Though it should be noted that in contrast to Harsevoort et al. [18], the present study included all patients who had hip pain, where none of them had osteogenesis imperfecta, which explains why this condition was not a risk factor in the present cohort. Furthermore, the present study examined possible risk factors for TOH, which were found to be male gender of middle age and left-side hip pain. Another study that was recently carried out in Jordan by Bashaireh et al. [21] examined the prevalence and risk factors of TOH over six years through retrospective analysis. The study detected 15 patients diagnosed with TOH. Furthermore, the patient’s average age was 41 years, with 14 males and only one female. Bashaireh et al. [21] showed that working as a healthcare professional is a risk factor for TOH.

An almost similar retrospective cohort study was carried out at Mayo Clinic, Rochester, Minnesota, over 15 years. The median age of 33 TOH patients was 47 years, and 20 patients were male. Out of 33 patients, 13 (39%) patients reportedly had a low bone mineral density. Contrary to the normal laboratory findings in the current study, the aforementioned study reported elevated levels of 24-hour urine calcium in two out of six, and low serum 25-hydroxyvitamin D (< 20 ng/mL) levels in five out of 12 TOH patients [13]. Although the present study did not include the employment and job of the included patients, the prevalence of the disease in Jordan is compatible with the prevalence in Saudi Arabia, with eight patients who have been diagnosed with TOH over four years in Saudi Arabia. Additionally, the present study demonstrated that the most affected part of the hip with TOH was the femur bone's neck, commonly the left side. Moreover, Trevisan et al. [22] examined TOH's risk factors in a small retrospective cohort of 23 patients. Around 65% of the patients were males. The common risk factors identified by Trevisan et al. [22] were smoking, male gender, previous episodes of TOH, and abnormal DEXA scanning. However, Trevisan et al. [22] recommended further investigations for the risk factors of TOH. Smoking more than 10 cigarettes/day was identified as the fourth most frequent risk factor for TOH in a study conducted among a retrospective cohort of TOH patients over 15 years [13]. Unfortunately, we did not find data on the smoking status of our patients.

Finally, the present study showed some limitations; due to the retrospective nature of the study, identifying risk factors is limited to only the data already available on the patient’s health records. Furthermore, this study was single centered, making the extrapolation of our findings to other centers inapplicable due to the limited external validity.

## Conclusions

TOH should always be explored in the differential diagnosis of acute hip pain in young males. The symptoms of TOH come on suddenly and may be very severe, but they go away over time if the patient avoids bearing weight as much as possible. The range of motion is typically maintained, with the exception of the rotation's most extreme points. Although the prevalence of TOH is not high, it represents a significant burden in older age, particularly among males. Future studies should consider a robust study design with a multicenter and prospective setting to further identify risk factors.
